# Evaluation of the IL-Phoenix chemistry electrolyte analyser

**DOI:** 10.1155/S1463924693000161

**Published:** 1993

**Authors:** F. Masiá, A. Alumá, C. Biosca, M. T. Easamajó, F. Antoja, R. Galimany

**Affiliations:** 1 Servei d'Anàlisis Clíniques Hospital Universitari ‘Germans Trias Pujol’ Carretera de Canyet Badalona s/n 08916 Spain; 2 Servei d'Anàlisis Clíniques Centre d'Assistència Primària ‘Dr Robert’ Plaça de la Medicina Badalona s/n 08911 Spain

## Abstract

This paper reports an evaluation of the IL-Phoenix Chemistry/Electrolyte Analyser; the evaluation was carried out in accordance with internationally recognized guidelines. The evaluation was performed in three steps: evaluation in routine conditions; assessment of interferences; and study of practicability. Seven constituents were studied under routine working conditions. Within-run imprecision rangedfrom 0.6% (CV) for chloride to 3.1% (CV) for glucose. Between-run imprecision ranged from 0.9% for sodium to 6.0% (CV) for urea. Sample-related carryover was not significant. The relative inaccuracy was acceptable; drift was negligible; linearity was agreed with the range showed by the supplier. Haemoglobin produced negative interferences with sodium and chloride. Turbidity interfered negatively with sodium, chloride, potassium and total calcium, andpositively with glucose. Bilirubin showed a negative interference with sodium, chloride and creatinine.


Journal of Automatic Chemistry, Vol. 15, No. 4 (July–August 1993), pp. 127–132

### Evaluation of the IL-Phoenix chemistry electrolyte analyser

F. Masià,[1] A. Alumà,[2] C. Biosca,[1] M. T. Casamajó,[2] F. Antoja[2] and R. Galimany[1]

[1] *Servei d'Anàlisis Clíniques, Hospital Universitari 'Germans Trias i Pujol', Carretera de Canyet s/n 08916 Badalona, Spain.* [2] *Servei d'Anàlisis Clíniques, Centre d'Assistència Primària 'Dr Robert', Plaça de la Medicina s/n 08911 Badalona, Spain*

*This paper reports an evaluation of the IL-Phoenix Chemistry/Electrolyte Analyser; the evaluation was carried out in accordance with internationally recognized guidelines. The evaluation was performed in three steps: evaluation in routine conditions; assessment of interferences; and study of practicability. Seven constituents were studied under routine working conditions. Within-run imprecision ranged from 0·6% (CV) for chloride to 3.1% (CV) for glucose. Between-run imprecision ranged from 0·9% for sodium to 6·0% (CV) for urea. Sample-related carry-over was not significant. The relative inaccuracy was acceptable; drift was negligible; linearity was agreed with the range showed by the supplier. Haemoglobin produced negative interferences with sodium and chloride. Turbidity interfered negatively with sodium, chloride, potassium and total calcium, and positively with glucose. Bilirubin showed a negative interference with sodium, chloride and creatinine.*

### Introduction

The IL-Phoenix is an eight-channel fully automated system that can analyse serum, plasma, urine and cerebrospinal fluid (CSF) samples. The IL-Phoenix utilizes potentiometric, spectrophotometric and amperometric techniques for *in vitro* measurements on stat samples, as well as routine runs.

The evaluation reported here was done according to the guidelines of the Comisión de Instrumentación de la Sociedad Española de Química Clínica [1]; the European Committee for Clinical Laboratory Standards [2]; and the Comission de Validation de Téchniques de la Société Française de Biologie Clinique [3]. The evaluation included an investigation of within-run and between-run imprecisions, sample-related carry-over, method comparison using patient specimens, drift, linearity and detection limit. The effects of an *in vitro* haemolysis, turbidity and bilirubin were also studied. The analyser was evaluated for system performance, the analytical procedure control, and ease of maintenance.

### Materials and methods

### *Instrument*

The IL-Phoenix (Instrumentation Laboratory, Lexington, Massachussetts, USA) is a fully automated eight-channel system that analyses serum, plasma, urine or CSF samples. The analyser utilizes potentiometric spectrophotometric and amperometric techniques for *in vitro* measurements on stat samples, as well as routine runs.

The analyser can be connected to a compatible data management system for retrieval and processing of patient data. The IL-Phoenix is available in two models:

(1) The Model 900 is used for analysis of sodium, potassium, chloride, glucose, urea, creatinine, total calcium and total carbon dioxide ($TCO_2$).

(2) The Model 905 performs as the 900, except that it measures total protein rather than $TCO_2$.

The evaluation was done with a Model 900; an examination of $TCO_2$ measurement was not included. Its performance with urine and CSF samples was not evaluated.

The IL-Phoenix can calculate osmolality, urea/creatinine ratio, creatinine clearance and anion gap. In addition, both models feature automatic linearity extension (ALEX), in which half the normal sample volume is aspirated if the sample results are outside the linear range of the analyser (except for $TCO_2$). The aspirated sample volume is diluted with an equal volume of distilled water.

A keyboard is used to specify the tests to be run, to start the analysis and to perform functions related to the tests; a colour monitor displays the tests to be run for each sample or retrieves data from previously run test and other performance functions. Up to three routine runs (30 samples/run) can be displayed in the monitor. Test results are printed automatically or upon request by the analyser dot-matrix printer.

The IL-Phoenix can handle 75 samples per hour, and up to 600 tests per hour. Stat tests are completed in 1 min.

The Model 900 requires 97 μl of sample for an eight-test panel (65 μl if Model 905).

A wide range of menu-selectable tests are available to confirm the status of analyser components. Analyser state messages, such 'Analysing' and 'Calibrating', are displayed. In addition, the analyser can display several attention messages.

Integrated fluidic circuits (manifolds) enable the use of short fluid paths for easier maintenance and avoid the leakage or mixture of fluids. Sodium, potassium, chloride and total calcium tests are run in the same manifold; another one is for glucose and urea; there is a third one for creatinine.

The working temperature is 37 °C for creatinine tests; 31 °C for gluocse and urea tests; and room temperature for the others.

0142–0453/93 $10.00 © 1993 Taylor & Francis Ltd.

*Methods*

The following methods are used:

(1) Sodium: ion selective electrode (Na glass).

(2) Potassium: ion selective electrode (neutral carrier membrane).

(3) Chloride: ion selective electrode (Ag/AgCl pellet).

(4) Total calcium: ion selective electrode (neutral carrier membrane).

(5) Glucose: immobilized glucose oxidase (EC 1.1.3.4) + amperometric oxygen electrode (fixed time reaction).

(6) Urea: immobilized urease (EC 3.5.1.5) + ammonium selective electrode (fixed time reaction).

(7) Creatinine: absorbance (Jaffe reaction) (rate).

The enzymes used in the glucose and urea assays are immobilized on the internal face of a nylon tube and the samples flow through it.

*Specimens and control solutions*

Forty serum samples, collected in vacuum tubes, were analysed. The control sera used were Control Key-trol Normal (ITC Diagnostics, lot No. 5020-6); and Control Key-trol Anormal (ITC Diagnostics, lot No. 5021-7). A sera pool from healthy individuals for the interferences studies.

*Reagents*

Reagents, buffers and calibrators are available exclusively from Instrumentation Laboratory. Reagents and buffers used were: IL Test ISE Buffer; IL Test ISE Reference; IL Test Glucose/Urea Buffer, IL Test Creatinine. The calibrators used were: Surecal 1 Calibrator (lot No. L0300089); Surecal 2 Calibrator (lot No. L0400128); Surecal 3 Calibrator (lot No. L0300090); Surecal 4 Calibrator (lot No. L0400117); Surecal 5 Calibrator (lot No. L0300082); Bilirubin Sigma B-4126 was used for the interferences studies. The Technicon CHEM-1 was used for the method comparison study.

*Evaluation protocol*

*Imprecision*

To study the within-run imprecision, 30 samples of control sera were tested at two levels in the same run. To evaluate the between-run imprecision, a further 20 samples at each of two levels were distributed in 20 different runs.

*Sample-related carry-over*

The carry-over caused by the sample was studied according to a model of Broughton [4]. Following an alternative permutation order, two control samples with different concentration were tested. Three high concentration specimens, followed by three low concentration specimens, were processed 20 times and the carry-over ratio $k$ was calculated. A mean for 20 determinations of $k$ was obtained. The carry-over ratio $c$ according to a model of Bennet *et al.* [5] was also calculated.

*Reagent-related carry-over*

Because of the performance mechanism of the IL-Phoenix the reagent-related carry-over was not studied.

*Method comparison*

Forty human sera (in different analytical series), covering the pathophysiological analytical range for each of the seven analytes, were analysed in parallel with the IL-Phoenix and the Technicon CHEM-1. The results obtained from both analysers were compared. The statistical evaluation was done by a non-parametric method according to Passing and Bablok [6,7].

*Drift*

A control (within the physiological range for each evaluated component) was assayed once immediately after calibration, and at eight and 12 hours after calibration. The study was repeated three times and the means of the obtained values were compared by the non-parametric test of Wilcoxon.

*Linearity*

Six decreasing dilutions of a serum for each evaluated analyte was prepared. Three aliquots of each dilution were assayed on three different days, to obtain nine sets of results.

*Detection limit*

The detection limit was taken as the mean of the least quantity or concentration of a substance that can be distinguished, with a given probability, from a reaction blank carried out under the same conditions. This ideal reaction blank was carried out using distilled water. The smallest concentrations of the analytes were obtained in three series of 10 determinations. The analytes studied were glucose, urea and creatinine.

*Imprecision and inaccuracy of the sample dilution system (Alex)*

Twenty samples of a pool with higher concentrations than the linear range of the analyser, for each evaluated analyte, were tested. Results were compared with the corresponding ones obtained by testing 20 samples of the same source solution diluted manually, according to a Student $t$ test for paired data.

*Interferences*

The effects of *in vitro* haemolysis, turbidity and bilirubin were evalauted on seven tests, according to the protocol of the Société Française de Biologie Clinique [3] and the NCCLS [8]. These potential interferents were studied by overloading a human sera pool with increasing concentrations of haemoglobin (up to 210 µmol), total bilirubin (up to 500 µmol/l) and triglycerides (up to 8·5 mmol/l).

*Table 1. Imprecision.*

| Test | | Within-run† | | | Between-run‡ | | |
|---|---|---|---|---|---|---|---|
| | | $\bar{x}$ | CV (%) | | $\bar{x}$ | CV (%) | |
| Total calcium | H | 3·01 | 0·9 | (1·5) | 2·94 | 2·0 | (1·6) |
| (mmol/l) | L | 1·87 | 1·3 | (1·5) | 1·80 | 2·0 | (1·6) |
| Chloride | H | 117 | 0·7 | (1·5) | 116 | 2·0 | (1·6) |
| (mmol/l) | L | 84 | 0·6 | (1·5) | 81 | 2·0 | (1·6) |
| Creatinine | H | 774 | 0·9 | (2·0) | 755 | 2·0 | (2·4) |
| (μmol/l) | L | 66 | 2·9 | (3·5) | 64 | 5·0 | (4·0) |
| Glucose | H | 16·1 | 1·0 | (1·0) | 16·2 | 2·6 | (1·6) |
| (mmol/l) | L | 3·3 | 3·1 | (2·5) | 3·3 | 3·0 | |
| Potassium | H | 7·4 | 0·9 | (1·5) | 7·3 | 1·2 | (1·6) |
| (mmol/l) | L | 3·1 | 1·0 | (2·0) | 3·1 | 2·6 | (2·0) |
| Sodium | H | 152 | 0·7 | (1·0) | 150 | 0·9 | (1·0) |
| (mmol/l) | L | 109 | 0·7 | (1·0) | 108 | 2·0 | (1·2) |
| Urea | H | 18·6 | 1·2 | (2·0) | 17·8 | 3·0 | (2·4) |
| (mmol/l) | L | 3·3 | 1·3 | (4·0) | 3·4 | 6·0 | (4·8) |

Figures in brackets are target values derived and described in reference [3].

The value obtained for each specimen and test was calculated as a percentage of the original concentration (before overloading) and expressed graphically.

### Results and discussion

*Imprecision*

Table 1 summarizes the results of the within-run and between-run imprecision studies. The acceptable coefficients of variation (CV %) are expressed according to the protocol of Validation de Techniques [3]. Within-run imprecision was acceptable for total calcium, chloride, creatinine, potassium, sodium and urea; for glucose within-run CV and between-run imprecision results, the obtained CVs were higher but near the tolerable limit.

*Sample-related carry-over*

The results of the study of the sample-related carry-over are shown in table 2. The mean of the *k* and *c* values were less than 2% (except the *c* value for urea).

*Method comparison with patient specimens*

The results of the relative inaccuracy study for each of the evaluated analytes are shown in table 3. A proportional difference ($p < 0{\cdot}05$) was detected between the two analytical methods for creatinine. There were also proportional and constant differences ($p < 0{\cdot}05$) for total calcium. For the remaining tests, the results reflected a good degree of agreement with the comparison instrument although *r* coefficients for sodium and chloride were slightly less than 0·800.

*Drift*

Drift was studied by processing the same specimen immediately after the calibration and 12 hours later, and the results were compared according to a non-parametric test of Wilcoxon for paired data. During the test, the specimen was stored at 4 °C. The drift was negligible; the results are shown in table 4.

*Linearity*

Linearity was within the range specified by the manufacturer; results are shown in table 5.

*Detection limit*

For a risk $\alpha = 5\%$ and large samples ($N \geq 30$), the upper limit of the blank confidence interval was calculated as being $\bar{x} + 2S$. This value that corresponds with the detection limit for the given risk – see table 6.

*Table 2. Sample-related carry-over.*

Sequence: H1 – H2 – H3 – L1 – L2 – L3
H = High, L = Low, $n = 20$

$$\text{Broughton: } K(\%) = \frac{L1 - L3}{H3 - L3} \times 100$$

$$\text{Bennett: } C(\%) = \frac{L1 - L3}{L3} \times 100$$

| | Concentration | | | Carry-over | |
|---|---|---|---|---|---|
| | H3 | L1 | L3 | $K(\%)$ | $C(\%)$ |
| Glucose (mmol/l) | 20·53 | 3·83 | 3·89 | −0·3 | −1·54 |
| Urea (mmol/l) | 23·00 | 3·89 | 3·74 | 0·79 | 4·08 |
| Creatinine (μmol/l) | 916·9 | 79·57 | 78··55 | 0·12 | 1·30 |
| Total calcium (mmol/l) | 3·69 | 2·12 | 2·13 | −0·90 | −0·65 |
| Sodium (mmol) | 166·2 | 111·6 | 111·7 | −0·18 | −0·08 |
| Potassium (mmol/l) | 8·24 | 3·16 | 3·16 | 0·07 | 0·12 |
| Chloride (mmol/l) | 122·1 | 86·23 | 86·15 | 0·20 | 0·08 |

*Table 3. Method comparison: Passing-Bablok regression.*

| | Range | (*r*) | *b* (i.c. 95%) | *a* (i.c. 95%) |
|---|---|---|---|---|
| Glucose (mmol/l) | 4·0–17·3 | 0·995 | 1·02 (1·00, 1·06) | −0·12 (−0·37, 0·00) |
| Urea (mmol/l) | 2·9–31·0 | 0·995 | 1·01 (0·95, 1·06) | −0·36 (−0·87, 0·033) |
| Creatinine (μmol/l) | 52·0–678·0 | 0·998 | 1·00 (0·97, 1.04) | −15·8* (−20·9, −11·2) |
| Total calcium (mmol/l) | 1·7–2·6 | 0·940 | 0·76* (0·70), 0·84) | 0·36* (0·16, 0·51) |
| Sodium (mmol) | 125–149 | 0·777 | 1·00 (0·83, 1·33) | 4·00 (−41·3, 26·5) |
| Potassium (mmol/l) | 2·3–5·6 | 0·887 | 1·02 ((0·88, 1·10) | 0·24 (−0·09, 0·81) |
| Chloride (mmol/l) | 95–119 | 0·753 | 1·00 (0·80, 1·33) | 2·00 (−33·0, 22·3) |

* Differences ($p < 0·05$) constant (A), proportional (B); $N = 40$.

*Sample dilution system*

A single analyte was used to check the correct performance of the automatic sample dilution system: creatinine. There were no statistically significant differences between automatic and manual dilution ($t = 0·62$; g.l. = 19; $P_{bilat} = 0·540$).

*Interferences*

The cut-off point in determining the interference level was fixed for each parameter according to the values reported in the protocol of the Société Française de Biologie Clinique [3]. Results are expressed in the figures as percentages. Haemoglobin produced a negative interference with sodium ([Hb] ≥ 15 μmol/l) and chloride ([Hb] ≥ 50 μmol/l) (see figure 1).

Turbidity caused by triglycerides (TG) produced a negative interference with sodium ([TG] ≥ 2·9 mmol/l, chloride ([TG] ≥ 3·1 mmol/l), potassium ([TG] ≥ 3·0 mmol/l), total calcium ([TG] ≥ 3·5 mmol/l); a positive interference with glucose ([TG] ≥ 5·1 mmol/l) was also shown by turbidity (figure 2).

Bilirubin (BR) showed a negative interference with sodium ([BR] ≥ 60 μmol/l), chloride ([BR] ≥ 60 μmol/l and creatinine ([BR] ≥ 200 μmol/l (figure 3).

*Table 4. Drift.*

| | After calibration | 12 h | % drift |
|---|---|---|---|
| Sodium (mmol/l) | 139·0 | 138·7 | −0·22 |
| Potassium (mmol/l) | 4·05 | 4·13 | 1·98 |
| Chloride (mmol/l) | 105 | 107 | 1·91 |
| Glucose (mmol/l) | 4·4 | 4·5 | 2·27 |
| Urea (mmol/l) | 4·7 | 4·9 | 4·26 |
| Creatinine (μmol/l) | 87 | 88 | 1·15 |
| Total calcium (mmol/l) | 2·40 | 2·42 | 0·83 |

$N = 3$.

*Table 5. Linearity.*

| Test | Supplier's range | Tested range |
|---|---|---|
| Sodium (mmol/l) | 100–180 | 80–160 |
| Potassium (mmol/l) | 1–9 | 1·5–9 |
| Chloride (mmol/l) | 70–130 | 60–130 |
| Glucose (mmol/l) | 0·5–25 | 0·5–25 |
| Urea (mmol/l) | 0·8–16 | 1–30 |
| Creatinine (μmol/l) | 0–1760 | 10–1700 |
| Total Calcium (mmol/l) | 1·25–3·75 | 1·04–3·75 |

*Practicability*

*Environmental factors*

The IL-Phoenix can be placed on a work table; it produces little noise and operates at room temperature. Buffers and reagents are stored at room temperature. It uses approximately 1 l of distilled water as a diluent for every 100 samples. The waste receptacle supplied does not have the capacity for the expected output of the analyser, although a larger one is available. No special conditions of humidity or electrical installation are required.

*Operator training*

For routine work, operator training is easy. The IL-Phoenix has a simple information program. The operator's guide is clear.

*Table 6. Detection limit.*

| | $\bar{x}$ | $S$ | $\bar{x} + 2S$ |
|---|---|---|---|
| Glucose (mmol/l) | 0·14 | 0·11 | 0·36 |
| Urea (mmol/l) | 0·45 | 0·07 | 0·59 |
| Creatinine (μmol/l) | 3·75 | 3·98 | 11·71 |

$N = 30$; risk $\alpha = 5\%$.

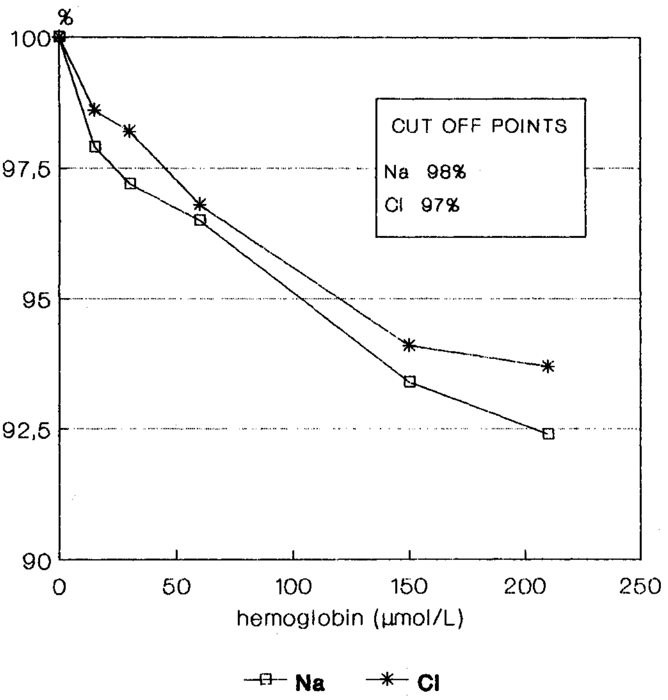

*Figure 1. Interferences: haemoglobin.*

*Flexibility*

The system is closed and the customer has to rely completely on the manufacturer. The methodologies cannot be modified. A bidirectional on-line connection is available.

*Work conditions*

The starting delay is 20 minutes (for calibration). The highest work speed is one sample every 55 s. The work is organized sequentially; however, it is possible to process

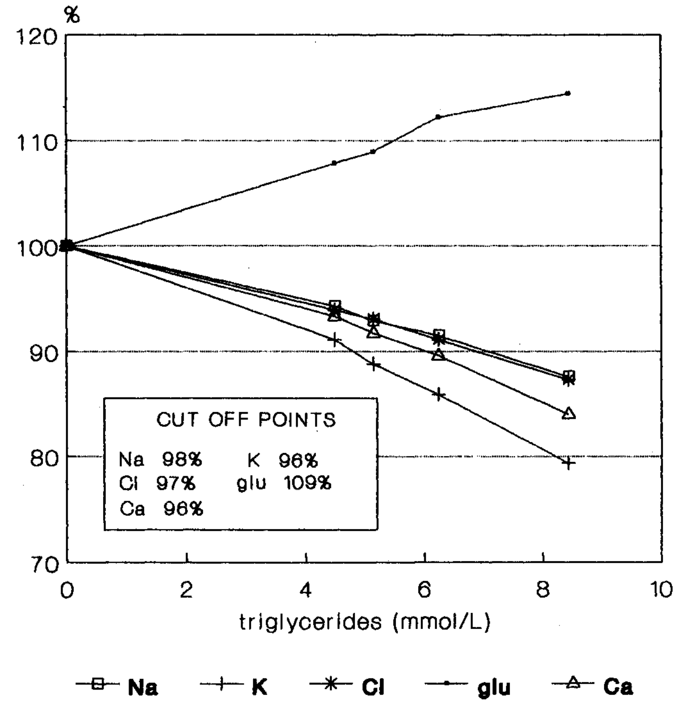

*Figure 2. Interferences: triglycerides.*

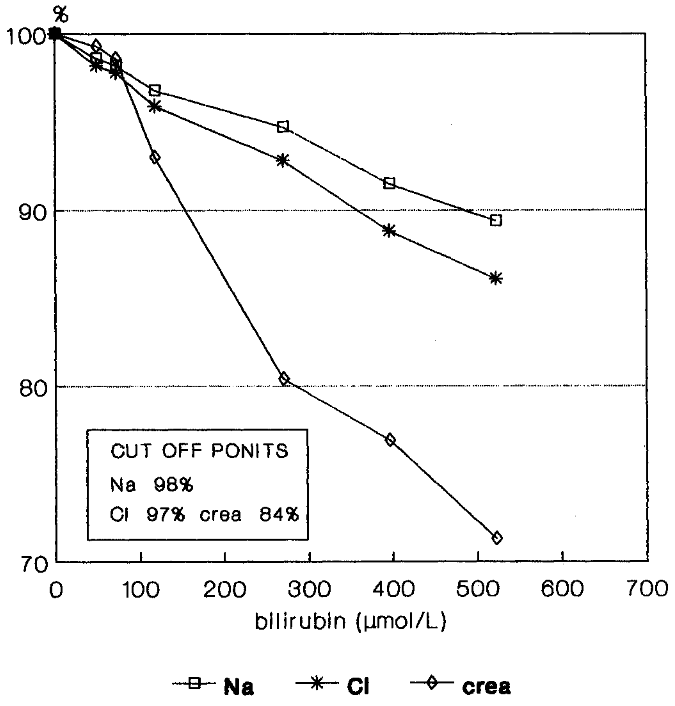

*Figure 3. Interferences: bilirubin.*

stat samples without interfering with the routine run. Neither primary blood sample tubes, nor bar-coded labels can be used. It is always possible to stop a routine run, but the analyser will complete the assay of the three following samples.

*Performance controls*

The IL-Phoenix is equipped with an alarm system that detects the buffer and reagent levels. It is possible to check the reaction temperatures and the motor or valves performance. The analyser is equipped with acoustic and screen alarms.

*Maintenance*

Maintenance operation is short and simple: 20 min weekly and 40 min monthly.

*Computer capabilities and reporting results*

The IL-Phoenix can report results in different formats. It is possible to get a new report of an old sample. Results are stored on a $3\frac{1}{2}$ in. diskette.

*Quality control*

The quality control system is able to use Westgard rules and Levy-Jennings charts.

### Conclusions

In routine conditions, within-run imprecision was good; between-run imprecision was acceptable. All the coefficients of variation were lower than 5% (except 6% for urea at low concentrations).

The relative inaccuracy study reflects a good agreement with the comparison instrument, except for chloride ($r = 0{\cdot}753$) and sodium ($r = 0{\cdot}777$); proportional and constant differences ($p < 0{\cdot}05$) have been shown for total calcium according to a Passing-Bablok regression.

No sample-related carry-over was found in the assayed analytes: values obtained are less than 2%. Drift was negligible; linearity agreed with the information provided by the supplier. Inaccuracy and imprecision on the automatic sample dilution system were good.

The following significant interferences were noted:

(1) Haemoglobin produced negative interferences with sodium and chloride.

(2) Turbidity interfered negatively with sodium, chloride, potassium and total calcium, and positively with glucose.

(3) Bilirubin showed a negative interference with sodium, chloride and creatinine.

The analyser performed acceptably during the evaluation study; the practicability of the system was good. Other tests are needed on the instrument to look at its usefulness in the emergency area of a laboratory.

### References

1. Comisión de Instrumentación del Comité Científico de la Sociedad Española de Química Clínica. Protocolo de evaluación de analizadores automáticos: evaluación de los módulos analíticos, Doc. E, ver. 3. *Bol. Inf. SEQC*, **34** (1986), 3–17.
2. European Committee for Clinical Laboratory Standards.—Guidelines for the Evaluation of Analysers in Clinical Chemistry, *ECCLS Document*, **3** (1986), 2.
3. Société Française de Biologie Clinique – Commission 'Validation de Techniques'. – Protocole de validation de techniques. *Ann. Biol. Clin.*, **44** (1986), 686–745.
4. Broughton, P., Carry-over in automatic analysers. *Journal of Automatic Chemistry*, **6** (1984), 94–95.
5. Bennet, A., Gartelmann, D., Mason, J. I. and Owen, J. A., Calibration, calibration drift and specimen interaction in autoanalyser systems. *Clinica Chimica Acta*, **29** (1970), 161–180.
6. Passing, H. and Bablok, W., A new biometrical procedure for testing the equality of measurements from two different analytical methods. Application of linear regression procedures for method comparison studies in Clinical Chemistry. *Journal of Clinical Chemistry and Clinical Biochemistry*, **21** (1983), 709–720.
7. Passing, H. and Bablok, W., Comparison of several regression procedures for method comparison studies and determination of sample size. Application of linear regression procedures for method comparison studies in Clinical Chemistry. Part II. *Journal of Clinical Biochemistry*, **22** (1984), 431–445.
8. National Committee for Clinical Laboratory Standards. Interference testing in Clinical Chemistry. *NCCLS Document EP7-P*, **6** (1986), 13.

